# A promoter haplotype of the *interleukin-18 *gene is associated with juvenile idiopathic arthritis in the Japanese population

**DOI:** 10.1186/ar1930

**Published:** 2006-03-17

**Authors:** Tomoko Sugiura, Nobuaki Maeno, Yasushi Kawaguchi, Syuji Takei, Hiroyuki Imanaka, Yoshifumi Kawano, Hisae Terajima-Ichida, Masako Hara, Naoyuki Kamatani

**Affiliations:** 1 Institute of Rheumatology, Tokyo Women's Medical University School of Medicine, 10-22 Kawada-cho, Shinjuku-ku, Tokyo, Japan; 2 Department of Infection and Immunity, Kagoshima University Graduate School of Medical and Dental Sciences, 8-35-1 Sakuragaoka, Kagoshima, Japan; 3 School of Health Sciences, Faculty of Medicine, Kagoshima University, 8-35-1 Sakuragaoka, Kagoshima, Japan; 4 Department of Pediatrics, Kagoshima University Graduate School of Medical and Dental Sciences, 8-35-1 Sakuragaoka, Kagoshima, Japan

## Abstract

Recently, we reported that genetic polymorphisms within the human *IL18 *gene were associated with disease susceptibility to adult-onset Still's disease (AOSD), which is characterized by extraordinarily high serum levels of IL-18. Because high serum IL-18 induction has also been observed in the systemic type of juvenile idiopathic arthritis (JIA), we investigated whether similar genetic skewing is present in this disease. Three haplotypes, S01, S02, and S03, composed of 13 genetic polymorphisms covering two distinct promoter regions, were determined for 33 JIA patients, including 17 with systemic JIA, 10 with polyarthritis, and 6 with oligoarthritis. Haplotypes were also analyzed for 28 AOSD patients, 164 rheumatoid arthritis (RA) patients, 102 patients with collagen diseases, and 173 healthy control subjects. The frequency of individuals carrying a diplotype configuration (a combination of two haplotypes) of S01/S01 was significantly higher in the JIA patients, including all subgroups, than in the healthy controls (*P *= 0.0045, Fischer exact probability test; odds ratio (OR) = 3.55, 95% confidence interval (CI) = 1.55–8.14). In patients with systemic JIA, its frequency did not differ statistically from that of normal controls. Nevertheless, it is possible that haplotype S01 is associated with the phenotype of high IL-18 production in systemic JIA because the patients carrying S01/S01 showed significantly higher serum IL-18 levels compared with patients with other diplotype configurations (*P *= 0.017, Mann-Whitney *U *test). We confirmed that the frequency of the diplotype configuration of S01/S01 was significantly higher in AOSD patients than in healthy control subjects (*P *= 0.011, OR = 3.45, 95% CI = 1.42–8.36). Furthermore, the RA patients were also more predisposed to have S01/S01 (*P *= 0.018, OR = 2.00, 95% CI = 1.14–3.50) than the healthy control subjects, whereas the patients with collagen diseases did not. In summary, the diplotype configuration of S01/S01 was associated with susceptibility to JIA as well as AOSD and RA, and linked to significantly higher IL-18 production in systemic JIA. Possession of the diplotype configuration of S01/S01 would be one of the genetic risk factors for susceptibility to arthritis in the Japanese population.

## Introduction

IL-18 is produced by a wide range of immune cells, such as monocytes, macrophages, and dendritic cells. Initially thought to be an IFN-γ-inducing factor of T cells and natural killer cells [1,2], IL-18 has been found to have multiple biological functions. Interestingly, IL-18 is a unique cytokine that stimulates both T helper (Th)1- and Th2-type immune responses, depending on its cytokine milieu [3]. In combination with IL-12, IL-18 induces IFN-γ production in Th1 cells, B cells, and natural killer cells, promoting Th1-type immune responses [4]. When cultured with IL-2, however, IL-18 induces Th2 lineage in CD4+ T cells [5]. In basophils and mast cells, IL-18 together with IL-3 also induces production of Th2 cytokines [6]. *In vivo*, IL-18 regulates innate and acquired immune responses, controlling either Th1 or Th2 cytokine balance. To date, inappropriate IL-18 production has been reported both in chronic inflammatory diseases such as Crohn's disease [7] and rheumatoid arthritis (RA) [8,9], and in allergic diseases such as bronchial asthma and atopic dermatitis [10].

We previously reported that serum IL-18 levels were significantly elevated and well correlated with disease activity and severity [11] in adult-onset Still's diseases (AOSD), which is characterized by high spiking fever, polyarthralgia, evanescent salmon-colored rash, liver dysfunction, splenomegaly and hyper ferritinemia [12]. Although serum levels of inflammatory cytokines are generally high in AOSD patients [13], the levels of IL-18 were enormously high, reaching more than 1,000 times the levels found in normal controls and other chronic inflammatory diseases such as RA [11,14]. Therefore, we have suggested that IL-18 is closely involved in the pathogenesis of AOSD.

Juvenile idiopathic arthritis (JIA) is a chronic arthritis of childhood and belongs to a group of clinically heterogeneous disorders including oligoarthritis, polyarthritis, systemic arthritis, secondary arthritis, and other arthritis types. According to the International League of Associations for Rheumatology (ILAR) classification criteria, oligoarthritis is further subdivided into persistent and extended oligoarthritis, and polyarthritis is further subdivided into rheumatoid factor-positive and negative polyarthritis. Thus, JIA is categorized into seven clinically distinct presentations [15], and the pathogenesis differs among the subgroups. Systemic JIA is characterized by systemic involvement, such as high spiking fever, skin rash, serositis and hepatosplenomegaly, and overproduction of inflammatory cytokines [16,17]. Maeno and colleagues [18] reported the serum levels of IL-18 were strikingly high in systemic JIA compared with other JIA subgroups and other childhood inflammatory disorders. Among systemic JIA patients, individuals with hepatosplenomegaly or serositis showed higher serum IL-18 levels than those without such manifestations [18]. In addition to similar clinical findings, aberrant production of IL-18 is a characteristic feature in both AOSD and systemic JIA, which is the reason why many investigators may consider these two diseases to be the same entity.

The human *IL18 *gene is located on chromosome 11q22.2 – q22.3 [19] and is composed of six exons; the translation-starting site is present in exon 2 [20]. It lacks a TATA box, and its expression is regulated by at least two distinct promoter regions, one of which is located upstream of untranslated exon 1 (promoter 1) [20-24], and the other upstream of exon 2 (promoter 2) [23]. Promoter 1 is up-regulated by various stimulants in macrophages [20], intestinal epithelial cells [21], and epithelial-like cell lines [24]. A 108 base-pair (bp) 5' flanking region upstream of exon 1 contains a *PU.1 *(purine-rich sequence) consensus-binding site and a GC-rich region; this region is critical for the sodium butyrate-stimulated promoter 1 activity [21,25]. Promoter 2 is considered to act constitutively [23], but regulation of human *IL18 *gene expression has not been fully examined.

We speculated that genetic polymorphisms within the promoter region of the *IL18 *gene might contribute to the high IL-18 production in AOSD. Thus, in the previous study, we performed a systematic search for polymorphisms in a 6.7 kb sequence, including a putative promoter region of the *IL18 *gene, and then identified 10 single nucleotide polymorphisms (SNPs) and a single 9 bp insertion [26]. The region had been reported to be upstream of exon 2 (intron 1) [24]. Later, Kalina and colleagues [20] determined that the 6.7 kb sequence was upstream of exon 1 instead of exon 2 by using 5' rapid amplification of cDNA ends; thus, this 6.7 kb region includes promoter 1 of the *IL18 *gene. We identified some of these polymorphisms as components of haplotypes and found there were three major haplotypes in the Japanese population (S01, S02, and S03). Furthermore, the frequency of haplotype S01, especially the diplotype configuration of S01/S01, was significantly higher in AOSD than in RA as well as in normal controls [26].

In the present study, we conducted a case-control study to evaluate possible associations of haplotype S01 of the *IL18 *gene with susceptibility to JIA, especially to systemic JIA.

## Materials and methods

### Patients and control subjects

The present study has been approved by the institutional Genome-Ethics Committee of Tokyo Women's Medical University and Kagoshima University. We examined 33 patients with JIA who were followed at the Pediatric Department of Kagoshima University Hospital, with informed consent. Of these patients, 17 had systemic JIA (female, 47.1%), 10 had polyarthritis (female, 70%), and 6 had oligoarthritis (female, 100%). All the patients satisfied the ILAR classification criteria for JIA [15]. We also examined 28 patients with AOSD (female, 79%), 164 patients with RA (female, 81%), and 102 patients with collagen diseases (female, 84%) who were followed at the Institute of Rheumatology, Tokyo Women's Medical University, with informed consent, as well as 173 healthy individuals (female, 64.7%). All the patients with AOSD met the criteria for AOSD of both Cush and colleagues [12] and Yamaguchi and colleagues [27]. All the RA patients fulfilled the 1987 classification criteria for RA of the American College of Rheumatology [28]. The patients with collagen diseases included 38 patients with systemic lupus erythematosus, 38 patients with scleroderma, and 26 patients with polymyositis/dermatomyositis. All the patients with these three diseases fulfilled the 1982 revised criteria of the American Rheumatology Association for the classification of systemic lupus erythematosus [29], the American College of Rheumatology criteria for scleroderma [30] and the diagnostic criteria of Bohan and Peter for polymyositis/dermatomyositis [31]. All subjects were Japanese.

### DNA isolation and genotyping

Genomic DNA was isolated from peripheral blood. For all the patients and control individuals, the genotype of SNPs 1, 2, 4, 5, 6, 9, 11, 12, 13, and 14 were determined by allelic discrimination chemistry using the ABI PRISM 7900HT Sequence Detection System (Applied Biosystems, Foster City, CA, USA). In the present study, we typed these 10 polymorphic sites for each individual because they were sufficient to estimate the haplotype and a diplotype configuration. As listed in Table 2, a set of forward and reverse primers and fluorescent-labeled probes were prepared. The probe designed to hybridize specifically to allele 1 was labeled by VIC, while that for allele 2 was labeled by FAM. A 10 μl portion of PCR mixture contained 9 pM each of the forward and reverse primers, 2 pM each of probes, 10 ng of genomic DNA as a template, and TaqMan PCR Universal Master Mix containing AmpliTaq Gold DNA polymerase (Applied Biosystems). During PCR, each probe annealed specifically to complementary sequences between the forward and reverse primer sites. AmpliTaq Gold DNA polymerase cleaved probes that hybridize to the target, and the cleavage separated the reporter dye from the probe. By comparing the fluorescent signals generated during PCR amplification, we determined the sequences present in the sample. When the fluorescent signal was VIC only, the sample was homozygous for allele 1. Similarly, with FAM fluorescence only, the sample was homozygous for allele 2. When both fluorescent signals were increased, the sample was heterozygous.

### Serum IL-18 measurement

Serum concentrations of IL-18 were measured by enzyme-linked immunosorbent assay (ELISA) with the use of a commercial kit (IL-18 ELISA Kit; MBL, Nagoya, Japan).

### Statistical analysis

To compare the frequencies of the haplotype or the diplotype configurations, we used the Fisher exact probability test. Differences were considered to be significant at *P *< 0.05. Odds ratios (ORs) were determined for disease susceptibility in carriers of a specific haplotype or a diplotype configuration. The 95% confidence intervals (CI) for ORs were also calculated. The data on serum IL-18 levels were expressed as the mean ± standard deviation, and the statistical significance of differences between two groups was determined by the Mann-Whitney *U *test.

## Results

### Polymorphisms of the human *IL18 *gene

**Figure 1:**
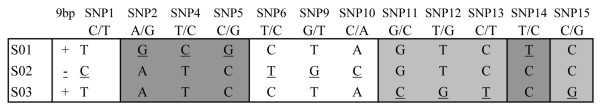
A unique combination of 13 polymorphisms comprising three different haplotypes: S01, S02 and S03. The 9 bp insertion, single nucleotide polymorphisms (SNPs) 1, 6, 9, and 10 (open box); SNP2, 4, 5, and 14 (dark gray box), and SNP11, 12, 13, and 15 (gray box) are in linkage disequilibrium. An underline indicates a minor allele in the normal Japanese population. +, 9 bp (AACAGGACA) is inserted; -, 9 bp is not inserted.

In the previous study, we have reported the presence of 10 SNPs (SNP1 to SNP10) and a single 9 bp (AACAGGACA) insertion in a 6.7 kb sequence upstream of exon 1 of the *IL18 *gene [26]. Of these, SNPs 1, 2, 4, 5, 6, 9, and 10 and the single 9 bp insertion are components of three major haplotypes, S01, S02, and S03, in the Japanese population. We also investigated previously reported SNPs by other investigators [22,32] within the *IL18 *gene and, with the use of a PENHAPLO computer program [33], found that some of them were also components of these haplotypes. SNP9 and SNP10 corresponded to SNPs previously reported by Giedraitis and colleagues [22] as -656 G/T and -607 C/A, respectively. They also reported three additional SNPs spanning from promoter 1 to exon 1. In this manuscript, we tentatively refer to -137 G/C as SNP11, +113 T/G as SNP12, and +127 C/T as SNP13. Furthermore, Kuruse and colleagues [32] reported three SNPs located upstream of exon 2, of which -920 T/C and -133 C/G were also components of three haplotypes, and are tentatively called SNP14 and SNP15, respectively. Thus, a single 9 bp insertion and SNPs 1, 2, 4, 5, 6, 9, 10, 11, 12, 13, 14, and 15 are the components of three haplotypes (Figure 1). The position of each SNP, allelic frequencies, and functions are summarized in Table 1. Nucleotides at the 12 SNP sites and presence or absence of the 9 bp insertion were as follows: haplotype S01, T, G, C, G, C, T, A, G, T, C, T, and C with the 9 bp insertion; haplotype S02, C, A, T, C, T, G, C, G, T, C, C, and C without the 9 bp insertion; haplotype S03, T, A, T, C, C, T, A, C, G, T, C, and G with the 9 bp insertion (Figure 1). The 9 bp insertion, and SNPs 1, 6, 9, and 10 were in complete linkage disequilibrium (D' = 1), while SNPs 2, 4, 5, and 14 were also in complete linkage disequilibrium (D' = 1). Furthermore, SNPs 11, 12, 13, and 15 were in complete linkage disequilibrium (D' = 1). These haplotypes spanned from the 5' 6.7 kb flanking exon 1 to intron 1, and included both promoter 1 and promoter 2 regions of the human *IL18 *gene.

### The frequencies of haplotypes and diplotype configurations in JIA

The allelic frequencies of three haplotypes of the *IL18 *gene in patients with JIA and healthy control subjects are summarized in Table 3. The frequency of haplotype S01 was significantly higher in the JIA patients as a whole than the healthy controls (*P *= 0.011, Fisher exact probability test; OR = 1.97, 95% CI = 1.16–3.35). With regard to the JIA subgroups, the patients with oligoarthritis were predisposed to have haplotype S01 (*P *= 0.0021, Fisher exact probability test; OR = 8.21, 95% CI = 1.77–38.04). In the polyarthritis and systemic subgroups, the frequency of haplotype S01 did not differ statistically from that of healthy controls. The frequency of haplotype S01 was also significantly higher in the patients with AOSD than in healthy controls (*P *= 0.028, Fisher exact probability test; OR = 1.89, 95% CI = 1.07–3.34). Furthermore, the patients with RA were also predisposed to have haplotype S01, compared with the healthy controls (*P *= 0.013, Fisher exact probability test; OR = 1.49, 95% CI = 1.10–2.02).

The six diplotype configurations in the Japanese population include S01/S01, S01/S02, S01/S03, S02/S02, S02/S03, and S03/S03. As shown in Table 4, the frequency of the S01/S01 diplotype configuration was significantly higher in the JIA patients than the healthy controls (*P *= 0.0045, Fischer exact probability test; OR = 3.55, 95% CI = 1.55–8.14). For the JIA subgroups, the frequency of the S01/S01 diplotype configuration was significantly higher in the patients with oligoarthritis (*P *= 0.0059, Fisher exact probability test; OR = 12.42, 95% CI = 2.15–71.55) and polyarthritis (*P *= 0.048, Fisher exact probability test; OR = 4.14, 95% CI = 1.09–15.75) than healthy controls. The frequency of S01/S01 in patients with systemic JIA did not differ statistically from that of healthy controls. In comparison with the normal controls, the frequency of S01/S01 also was significantly higher in the AOSD (*P *= 0.011, Fisher exact probability test; OR = 3.45, 95% CI = 1.42–8.36) and RA patients (*P *= 0.018, Fisher exact probability test; OR = 2.00, 95% CI = 1.14–3.50).

### Association of the S01/S01 diplotype configuration with serum IL-18 levels

**Figure 2:**
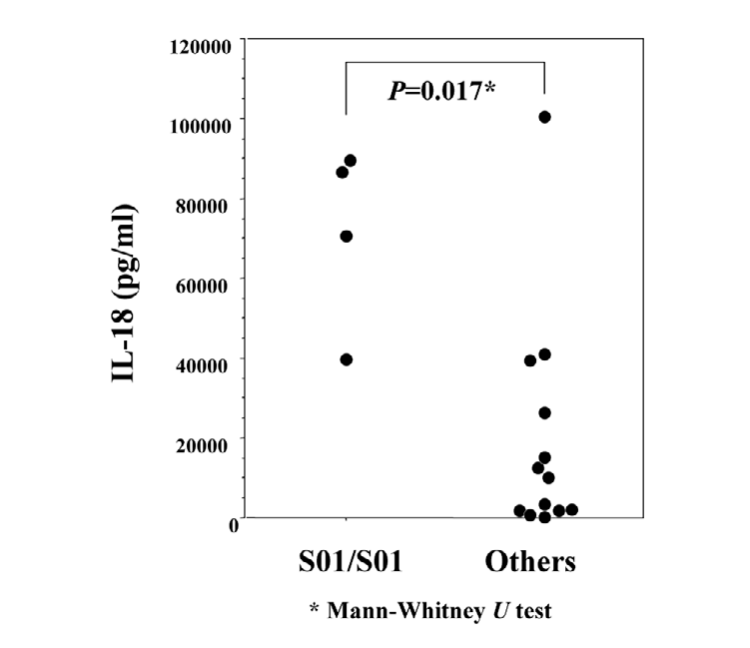
Serum levels of IL-18 in systemic juvenile idiopathic arthritis (JIA) patients with the S01/S01 diplotype configuration (*n* = 4) and other diplotype configurations (*n* = 13).

Serum IL-18 levels were measured for each patient with JIA before immunosuppressive treatment. As reported previously [18], serum IL-18 levels were enormously high in patients with systemic JIA compared to other subgroups of JIA. In the 17 patients with systemic JIA, serum IL-18 levels were significantly higher in the patients carrying S01/S01 (*n* = 4) than in those carrying other diplotype configurations (*n* = 13) (78,683 ± 22,778 pg/ml versus 10,122 ± 28,210 pg/ml; *P *= 0.017 by Mann-Whitney *U *test; Figure 2). In the other JIA subgroups, mean IL-18 levels in patients with and without the S01/S01 diplotype configuration were 572 ± 327 pg/ml versus 325 ± 302 pg/ml in those with polyarthritis, and 252 ± 124 pg/ml versus 219 ± 48 pg/ml in those with oligoarthritis. The IL-18 levels did not differ statistically among the diplotype configurations in the patients with polyarthritis and oligoarthritis.

## Discussion

The present study has demonstrated a strong association between the haplotype S01 of the *IL18 *gene and JIA as well as AOSD and RA in the Japanese population. Initially, we speculated that haplotype S01 is specific to systemic JIA because of previous studies that showed enormously high IL-18 production both in AOSD and systemic JIA [11,14,18] and genetic skewing of the haplotype S01 in AOSD [26]. However, haplotype S01 was associated with all the subgroups of JIA in the Japanese population, and we did not find the expected statistically significant correlation of haplotype S01 with susceptibility to systemic JIA. Furthermore, haplotype S01 was found to be associated with the whole group of arthritis diseases, including JIA, ASOD and RA.

Genetic polymorphisms of the human *IL18 *gene have been associated with a wide variety of diseases, including allergic diseases and inflammatory diseases [32,34-36]. These observations might reflect the redundancy of the IL-18 protein, which has multiple biological functions promoting both Th1- and Th2-type immune responses. Previously reported polymorphic sites concentrated on two promoter regions and the untranslated exon 1 of the *IL18 *gene. Most of the investigators have compared the frequency of each allele in the disease population with that of a healthy population. In a previous study [26], we demonstrated that all of these genetic polymorphisms comprised a haplotype. We therefore checked the genotypic data reported by other investigators and estimated the carrying rate of each haplotype in the disease populations. Interestingly, we found differently genetically skewed haplotypes in each disorder. For example, in the white population of Germany, patients with atopic phenotypes were predisposed to have -137 C in promoter 1, +113 G in exon 1, +127 T in exon 1, and -133 G in promoter 2 [32], and each allele corresponded to the minor allele of SNPs 11, 12, 13, and 15, respectively. All of these were the components of haplotype S03. Other investigators reported that SNPs specific for haplotype S03 are linked to susceptibility to atopic eczema in Germany [34], and asthma in Japanese [35]. It was likely that haplotype S03 of the *IL18 *gene was associated with atopic disorders, typical of Th2-dominant disease. On the other hand, Japanese patients with sarcoidosis were reported to be associated with polymorphisms specific for haplotype S02 [36]. The present study shows that haplotype S01 is associated with JIA as well as AOSD and RA in the Japanese population, with each having arthritis as a phenotype. In view of imbalanced immune responses, these diseases may be considered as Th1-dominant disorders [37-39]. By analyzing haplotypes, it became clear that haplotypes S01 and S03 of the *IL18 *gene are involved in the susceptibility of quite different types of disorders.

It is important to note there is an essential racial difference in the distribution of haplotypes. According to our data, the frequency of haplotype S01 of the *IL18 *gene is 37.9% and that of the S01/S01 diplotype configuration is 13.9% in the Japanese population. We also estimated the frequency of haplotypes and/or the diplotype configurations in the different ethnic groups of previous reports. The frequency of the S01/S01 diplotype configuration was considered to be only 1% in the healthy Swedish population [22] and only 0.05% in the Chinese population [38], much lower than that of the Japanese population. Also, in German whites, the frequency of haplotype S01 was 13% [40]. Interestingly, the existence of the fourth major haplotype, which we tentatively call haplotype S04, was reported in the Polish population [41]. The haplotype S04 is composed of combinations of nucleotides of SNP 10 C and SNP 11 C [41]; we have never observed this haplotype in the Japanese population. As described, the carrying rate of each haplotype is quite different among ethnic groups, which would be one reason why a disease-associated haplotype is not necessarily common among them. Indeed, no increase in the frequency of the S01/S01 diplotype configuration was observed in RA patients in Chinese patients [42] and in JIA patients in Germany [40]. One reason why haplotype S01 of the *IL18 *gene is skewed in Japanese arthritis patients is due to a basal high frequency of the haplotype S01 in the Japanese population.

The findings in the present study raise the question of how these genetic polymorphisms within the human *IL18 *gene influence the phenotype. One consideration is that nucleotide substitution directly influences the transcription of the gene. SNP11 is located at the GATA3 binding site, which is involved strongly in Th2 differentiation [43]. The C allele of SNP11 is specific to the haplotype S03, which might be a good explanation for the association of haplotype S03 and atopic disorders. As for haplotype S01, there have been few good explanations as to why it is linked to extraordinarily high IL-18 production. Nucleotide substitutions specific for haplotype S01 (SNP2, 4, 5 and 14) do not create known nucleotide factor-binding sequences; however, it is still possible that undefined genetic polymorphisms in linkage disequilibrium with haplotype S01 exist in other regions of the *IL18 *gene and influence the expression of IL-18 protein.

The aim of the present study was to examine whether haplotype S01 of the *IL18 *gene was skewed in systemic JIA. The frequency of the S01/S01 diplotype configuration seemed to be higher in patients with systemic JIA than in healthy controls (23.5% versus 13.9%), but the difference was not statistically significant, probably because of the relatively small study populations. Although we could not prove haplotype S01 was associated with disease susceptibility, the haplotype was linked to some clinical features. The patients carrying the S01/S01 diplotype configuration showed significantly higher IL-18 protein levels than those without it (*P *= 0.017). Thus, it is possible that haplotype S01 of the *IL18 *gene is linked to the phenotype of high IL-18 production. Because serum IL-18 levels indicate disease severity [18], the haplotype S01 might be a marker of severe disease condition in systemic JIA. In addition, although the differences were not statistically significant, the patients with the S01/S01 diplotype configuration tended to be younger at disease onset, need immunosuppressive drugs in addition to high doses of corticosteroids to control severe disease activity, and have the complication macrophage-activating syndrome (data not shown). Similarly, in AOSD, the S01/S01 diplotype configuration correlated with disease severity (unpublished data).

The mechanism of induction of enormously high IL-18 protein production in AOSD and systemic JIA is unclear, but it is likely that infectious agents such as viruses trigger the activation of macrophages and induce IL-18 production, and that some immunological breakdowns fail to control sustained activation of macrophages and consequently cause continuous IL-18 production. Several candidate genes would be involved in these phenomena besides the *IL18 *gene itself. They might include factors regulating IL-18 mRNA transcription or translation and caspase-1, which cleaves pro-IL-18 into a mature form [44]. We suggest that haplotype S01 of the *IL18 *gene might influence IL-18 protein production via unknown mechanisms, which explains in part the enormously high IL-18 production in systemic JIA. Furthermore, homozygosity for S01 would be more important for susceptibility to JIA as well as AOSD and RA than only carrying the S01 haplotype, since the *P* value for the former was smaller than that of the latter.

The present study also shows that haplotype S01 of the *IL18 *gene is widely linked to susceptibility to arthritis in the Japanese population. As described above, IL-18 is a key cytokine involved in the pathogenesis of both AOSD and systemic JIA [11,14,18]. High IL-18 protein could induce liver injury [45] or arthritis [8]. In RA, several lines of evidences suggest that IL-18 plays a role in the pathogenesis because IL-18 is up-regulated and induces production of inflammatory cytokines such as tumor necrosis factor-alpha in synovium [8,9]. In collagen-induced arthritis, IL-18 promotes arthritis via tumor necrosis factor-alpha induction [46]. In another study, IL-18 knockout mice showed a reduced degree of inflammation, and the administration of recombinant IL-18 reversed collagen-induced arthritis [47]. In these disorders, including JIA, AOSD and RA, overproduction of IL-18, probably together with IL-12, would shift the immune response to the Th1 lineage. Indeed, peripheral blood obtained from AOSD patients showed more IFN-γ producing Th cells and a higher Th1/Th2 ratio than in healthy controls [37]. Th1 cytokine expression has been demonstrated in the synovium of RA and JIA [38,39]. As described, IL-18 would be involved in the pathogenesis of arthritis directly or via production of other cytokines, and probably would promote a Th1-type immune response. Among three haplotypes of the *IL18 *gene, haplotype S01 might contribute genetically to the development of arthritis in the Japanese population. This is supported by the observation that the frequency of the S01/S01 diplotype configuration was significantly higher in arthritis patients as a whole, including JIA, AOSD and RA, than normal controls (*P *= 0.0013, OR = 2.36, 95% CI = 1.36–4.12) or patients with collagen diseases (*P *= 0.0069, OR = 2.39, 95% CI = 1.22–4.75, data not shown).

## Conclusion

The frequency of haplotype S01 of the *IL18 *gene, which has been shown to be the genetic risk factor for susceptibility to AOSD, is also high in Japanese patients with JIA. The diplotype configuration of S01/S01 further increases the risk for the disease susceptibility. Although the frequency of the S01/S01 diplotype configuration did not differ statistically from that of normal controls in patients with systemic JIA, individuals carrying this diplotype configuration showed significantly higher serum IL-18 levels. The skewing of haplotype S01 and the S01/S01 diplotype configuration was also observed in patients with RA as well as in those with AOSD. In conclusion, having the S01/S01 diplotype configuration in the *IL18 *gene would be one genetic risk factor for susceptibility of the Japanese population to JIA, as well as RA and AOSD, and might contribute to high IL-18 protein production in systemic JIA.

## Abbreviations

AOSD = adult-onset Still's disease; bp = base-pair; CI = confidence interval; IFN = interferon; IL = interleukin; ILAR = International League of Associations for Rheumatology; JIA = juvenile idiopathic arthritis; OR = odds ratio; RA = rheumatoid arthritis; SNP = single nucleotide polymorphism; Th = T helper.

## Competing interests

The authors declare that they have no competing interests.

## Authors' contributions

TS conceived the study and drafted the manuscript. NM was responsible for the recruitment and classification of patients, performed ELISA, and helped to draft the manuscript. Y Kawaguchi participated in the design and coordination of the study, and recruited a subset of the patients. ST conceived the study together with Y Kawano and participated in the design of the study. HI and HTI recruited a subset of patients. MH recruited a subset of patients and participated in coordination of the study. NK participated in the design and coordination of the study. All authors read and approved the final manuscript.

## Figures and Tables

**Table 1:** Summary of polymorphisms comprising the three haplotypes

Call of SNP	Genotype allele 1/2 (frequency of allele2^a^)	JSNP-ID	Location	Function	Previously reported call (reference no.)
9 bp	-/+ (0.52)	ssj 0008978	5' flanking	Unknown	
SNP1	C/T(0.52)	ssj 0008979	5' flanking	Unknown	
SNP2	A/G(0.38)	ssj 0008980	5' flanking	Unknown	
SNP4	T/C(0.38)	ssj 0008982	5' flanking	Unknown	
SNP5	C/G(0.38)	ssj 0008983	5' flanking	Unknown	
SNP6	T/C(0.52)	ssj 0008984	5' flanking	Unknown	
SNP9	G/T(0.52)	ssj 0008987	5' flanking	Unknown	-656G/T (22)
SNP10	C/A(0.52)	ssj 0008988	5' flanking	CRE	-607C/A (22)
SNP11	G/C(0.14)	ssj 0008989	5' flanking	GATA-3	-137G/C (22)
SNP12	T/G(0.14)	ssj 0008990	Exon 1	Untranslated	+113T/G (22)
SNP13	C/T(0.14)	ssj 0008991	Exon 1	Untranslated	+127C/T (22)
SNP14	T/C(0.62)	ssj 0008992	Intron 1	Unknown	-920T/C (32)
SNP15	C/G(0.14)	ssj 0008993	Intron1	NF-1	-133C/G (32)

^a^The frequency of allele 2 in a normal Japanese population (*n* = 173). 9 bp, 9 base-pair insertion; CRE, cyclic AMP-responsive element-binding protein; GATA-3, GATA-binding protein 3; NF-1, nuclear factor-1. JSNP, a database of Japanese Single Nucleotide polymorphisms [48].

**Table 2:** Primers and probes for used allelic discrimination chemistry

Forward and reverse primers (5' to 3')	Fluorescent-labeled probes (5' to 3')
SNP1F GCCCAGCTAATTTTGTTTTGTTTTGTT	SNP1 allele1 VIC- TTGCAGCGTTGCCCA-MGB
SNP1R GGAGTATCGTTTGAGGCTAAGAGTT	SNP1 allele2 FAM- TATTGCAGTGTTGCCCA-MGB
SNP2F TCCTAGCTCTGGGCATACGAAT	SNP2 allele1 VIC-CCACTTATCTATAGAGCTT-MGB
SNP2R AGCTGTATGCCTATGGAGCCTTA	SNP2 allele2 FAM-CACTTATCTGTAGAGCTT-MGB
SNP4F CTGGCAATTCTGCCTTGTTTCAG	SNP4 allele1VIC- CCGAAGATAAAAGAATC-MGB
SNP4R GGATTACAGCCGTGAGCCA	SNP4 allele2 FAM- CGAAGATAGAAGAATC-MGB
SNP5F AAGCTGAGGCAGGAAGATCAC	SNP5 allele1 VIC- TCGGGAGTTCGAGACC-MGB
SNP5R ACACAGGGTTTCACCATGCT	SNP5 allele2 FAM- CGGGAGTTGGAGACC-MGB
SNP6F TCCCTACTGTTGTTTCCGCC	SNP6 allele1VIC-TGAAGACCCTGGGC-MGB
SNP6R CCCGAAGTCCGAGCACC	SNP6 allele2 FAM-TGAAGACCCCGGGC-MGB
SNP9F GAAAGTTTTAACACTGGAAACTGCAA	SNP9 allele1 VIC-TTTTGGTAGCCCTCTC-MGB
SNP9R TTACACTTTCTGCAACAGAAAGTAAGC	SNP9 allele2 FAM-TTTTGGTATCCCTCTCC-MGB
SNP11F ACAGGTTTTGGAAGGCACAGA	SNP11 VIC- ACGGAAGAAAAGATTT-MGB
SNP11R AATAAAGTGGCAGAGGATACGAGTACT	SNP11 FAM-ACGGAAGAAAACATTT-MGB
SNP12F AATTGTCTCCCAGTGCATTTTGC	SNP12 allele1 VIC-CTGCCAACTCTGGCTG-MGB
SNP12R TCTTCCCGAAGCTGTGTAGACT	SNP12 allele2 FAM- CCAACGCTGGCTG-MGB
SNP13F AATTGTCTCCCAGTGCATTTTGC	SNP13 allele1 VIC-CAGCCGCTTTAGC-MGB
SNP13R TCTTCCCGAAGCTGTGTAGACT	SNP13 allele1 FAM- CAGCCACTTTAGC-MGB
SNP14F AGTTTAAACCATGTCTCAAGATCTCTGC	SNP14 allele1 VIC-TTGTGCTACAATTATT-MGB
SNP14R AGTACTTGTGACTCTGTCATTAATAGAAATACCT	SNP14 allele2 VIC-TTGTGCTACAGTTATT-MGB

**Table 3:** Numbers and frequencies of haplotype S01, S02 and S03

	S01 (%)	*P*^a^	S02 (%)	S03 (%)
Normal	131 (37.9)	-	165 (47.6)	50 (14.4)
JIA				
All	36 (54.5)	0.011	25 (37.9)	5 (7.6)
Oligoarthritis	10 (83.3)	0.0021	2 (16.7)	0
Polyarthritis	10 (50.0)	NS	7 (35.0)	3 (25.0)
Systemic	16 (47.1)	NS	16 (47.1)	2 (5.9)
AOSD	30 (53.6)	0.028	20 (35.7)	6 (10.7)
RA	156 (47.6)	0.013	119 (36.3)	53 (16.2)
Collagen diseases	87 (42.6)	NS	89 (43.6)	28 (13.7)

^a^The frequency of S01 was compared with that of normal subjects and analyzed by Fisher exact probability test. AOSD, adult-onset Still's disease; JIA, juvenile idiopathic arthritis; NS, not significant; RA, rheumatoid arthritis.

**Table 4:** Numbers and frequencies of diplotype configurations

	S01/S01 (%)	*P*^a^	Other types^b ^(%)
Normal	24 (13.9)	-	149 (86.1)
JIA	12 (36.4)	0.0045	21 (63.6)
All			
Oligoarthritis	4 (66.7)	0.0059	2 (33.3)
Polyarthritis	4 (40.0)	0.048	6 (60.0)
Systemic	4 (23.5)	NS	13 (76.5)
AOSD	10 (35.7)	0.011	18 (64.3)
RA	40 (24.4)	0.018	124 (75.6)
Collagen diseases	14 (13.7)	NS	88 (86.3)

^a^The frequency of the S01/S01 diplotype configuration was compared with that of normal subjects and analyzed by Fisher exact probability test. ^b^Other types include the diplotype configurations S01/S02, S01/S03, S02/S02, S02/S03 and S03/S03. AOSD, adult-onset Still's disease; JIA, juvenile idiopathic arthritis; NS, not significant; RA, rheumatoid arthritis.
